# Design and Optimization of an Adaptive Knee Joint Orthosis for Biomimetic Motion Rehabilitation Assistance

**DOI:** 10.3390/biomimetics9020098

**Published:** 2024-02-07

**Authors:** Kun Liu, Shuo Ji, Yong Liu, Shizhong Zhang, Lei Dai

**Affiliations:** School of Mechanical and Aerospace Engineering, Jilin University, Changchun 130025, China; jishuo20@mails.jlu.edu.cn (S.J.); liuyong19@mails.jlu.edu.cn (Y.L.); zsz21@mails.jlu.edu.cn (S.Z.); dailei21@mails.jlu.edu.cn (L.D.)

**Keywords:** knee joint orthosis, biomimetic motion, rehabilitation assistance, structural optimization

## Abstract

In this paper, an adaptive knee joint orthosis with a variable rotation center for biomimetic motion rehabilitation assistance suitable for patients with knee joint movement dysfunction is designed. Based on the kinematic information of knee joint motion obtained by a motion capture system, a Revolute-Prismatic-Revolute (RPR) model is established to simulate the biomimetic motion of the knee joint, then a corresponding implementation for repetitively driving the flexion–extension motion of the knee joint, mainly assembled by a double-cam meshing mechanism, is designed. The pitch curve of each cam is calculated based on the screw theory. During the design process, size optimization is used to reduce the weight of the equipment, resulting in a reduction from 1.96 kg to 1.16 kg, achieving the goal of lightweight equipment. Finally, a prototype of the designed orthosis with the desired biomimetic rotation function is prepared and verified. The result shows that the rotation center of the prototype can achieve biomimetic motion coincident with the rotation center of an active knee joint, which can successfully provide rehabilitation assistance for the knee joint flexion–extension motion.

## 1. Introduction

The incidence rate of stroke has remained high over the past decades [1]. Stroke is a neurological disorder characterized by motor dysfunction, especially knee joint motion dysfunction, which seriously affects the quality of life of patients [2]. The number of people with limb injuries caused by traffic accidents is also increasing, leading to a gradual increase in the number of patients with motor dysfunctions [3,4]. For patients with motor dysfunction, long-term scientific biomimetic rehabilitation training can effectively improve physical motion ability and promote the recovery of the nervous system. Biomimetic rehabilitation training means that the motion mode of the rehabilitation equipment is the same as that of other people [5,6]. Artificial assistance for patients undergoing motion rehabilitation training is a complex and professional task that requires a lot of physical energy and takes a long time. Therefore, in order to reduce the workload of rehabilitation therapists, developing scientific and effective personalized motion rehabilitation equipment is a meaningful task. Biomimetic rehabilitation equipment can adaptively assist in human joint movement, thereby improving the comfort of patients during the rehabilitation process. The knee joint is one of the most complicated joints in the human body; its flexion and extension is not a single rotational movement but a combination of rotational movement and sliding movement [7,8]. The development of biomimetic knee joint assistance equipment can effectively improve both the scientific nature and comfort of knee joint rehabilitation training for patients.

In recent years, there have been many proposals for rehabilitation equipment to assist knee joint movement [9]. Common lower limb rehabilitation exoskeleton devices are equipped with motors on the outer side of the knee joint to drive knee joint rotation. However, the rotor of the motor and the rotation center of the knee joint cannot overlap in real time, resulting in poor experience and rehabilitation effect [10,11]. To improve the comfort of patients during the rehabilitation process, a lower limb exoskeleton for the knee joint motion with two degrees of freedom was designed in [12]. The parasitic force in the shank between the exoskeleton and the human body was used as the control signal of the controller and the joint trajectories of exoskeleton were adjusted to minimize the parasitic force. While this greatly improved the comfort of the patient, the knee joint rotation driven by the exoskeleton remained different from the physiological rotation required by the knee joint. A knee exoskeleton with an adaptive instantaneous rotation center and impact absorption was developed in [13], which contained a cross-configuration mechanism adapted to the time-varying rotation center of the active knee joint. However, the structure of the exoskeleton was complex and difficult to manufacture. HUMA is an electricity-powered lower-limb exoskeleton in which the structure of the knee joint consists an actuator and double four-bar linkages [14]. The first four-bar linkage is responsible for transmitting power, while the second is designed to implement polycentric knee motion to realize biomimetic movement at the knee joint. However, the exoskeleton contains many parts that it is difficult to assemble. ACJ has an adaptive trajectory of the rotation center that can automatically match the instantaneous rotation center of the attached knee joint; however, the complex structure of the equipment leads to difficulties in later maintenance [15]. Therefore, knee joint assistance devices with biomimetic motion performance can play an important role in knee joint rehabilitation exercise. This paper presents the design of an adaptive knee joint assistance mechanism with biomimetic motion effects based on the trajectory of the instantaneous rotation center of the knee joint during flexion and extension.

As a wearable human motion assistance device, in addition to its complete functionality, the light weight of the structure is very important to help improve the portability of the device and effectively reduce the manufacturing cost. Using the finite element analysis method to reduce the mass of equipment is a common method [16,17]. For example, the finite element software was used in [11] to reduce the mass of the motor fixing mechanism and thigh brace of the wearable exoskeleton robot, which greatly reduced the material consumption and optimized the structural shape. The weight of the chain in exoskeleton equipment can be reduced through topology optimization, which has made an important contribution to reducing the weight of exoskeleton equipment [18]. In [19], the finite element model of multifunctional rehabilitation equipment was established using finite element software and the goal of lightweight equipment was realized by size optimization. Topology optimization was used in [20] to reduce the mass of the splint in a finger orthosis, with its mass being successfully reduced by 30.52%. Therefore, it is necessary to use the finite element method for weight analysis of the designed mechanical structure.

In this paper, an adaptive knee joint orthosis for biomimetic motion assistance is developed that can be used to assist human knee joint extension in sitting position. Compared with traditional knee joint auxiliary equipment, the rotation center of the equipment can achieve biomimetic rotation coincident with the rotation center of an active knee joint. Based on knee joint motion experiments, the knee joint kinematics are analyzed and modeled in detail. Then, the mechanical structure of the knee joint orthosis is designed and optimized by finite element analysis.

## 2. Analysis of Knee Joint Motion Characteristics

**Figure 1:**
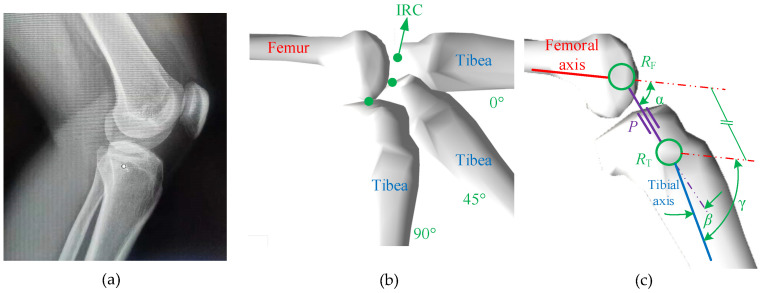
The RPR biomimetic knee joint model: (**a**) the position of the bones in the knee joint, (**b**) the position of the instantaneous rotation center (IRC) of the knee joint on the sagittal plane of the human body, and (**c**) the RPR model.

The knee joint is one of the most complex joints in the human body, consisting of the lower femur, upper tibia, patella, related muscles, and cartilage [21]. The position of the bones in the knee joint is shown in Figure 1a. During the flexion–extension rotation, the tibia always rotates around the femur based on a series of instantaneous axes perpendicular to the sagittal plane of the human body. The position of the instantaneous rotation center (IRC) in the sagittal plane of the human body is time-varying, as shown in Figure 1b. Under the premise of only considering the motion of the knee joint in the sagittal plane of the human body, a Revolute-Prismatic-Revolute (RPR) model is established to characterize the rotation of a knee joint, which includes two rotating pairs *R*_F_, *R*_T_ and a moving pair *P*, as shown in Figure 1c.

The relationship between the relevant parameters in the RPR model is as follows:

(1) $$\left\{\begin{array}{c} \alpha +\beta =\gamma \\ P=L(\gamma ) \end{array}\right.,$$

where *α* is the angle between the slip line of the moving pair and the femoral axis, *β* is the angle between the slip line of the moving pair and the tibial axis, *γ* is the knee joint angle between the tibial axis and the femoral axis, *P* is the length of the moving pair, and *L* is the function of the length of the moving pair with respect to the knee joint angle.

## 3. Knee Joint Movement Experiment

**Figure 2:**
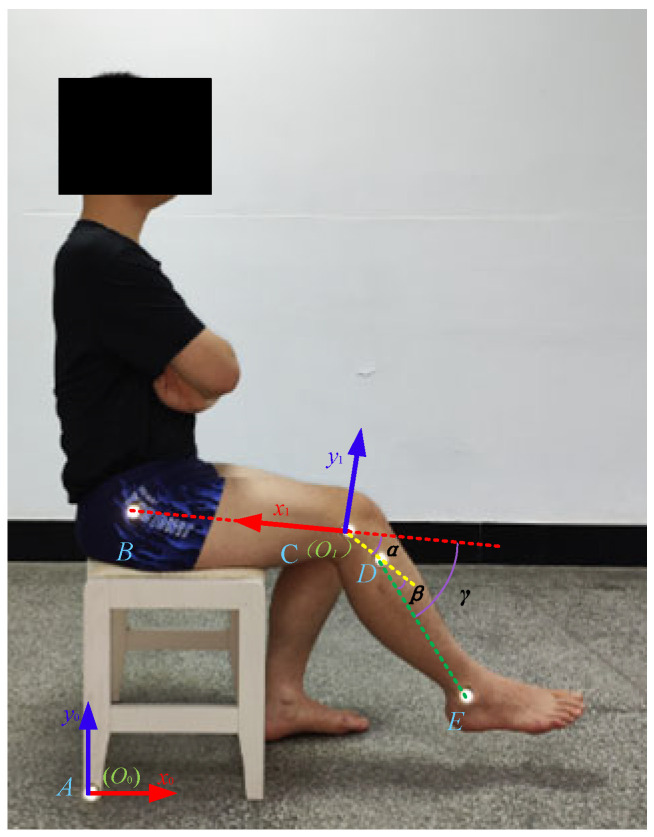
The knee joint extension process of the first subject, with markers.

The Vicon (Vicon Motion Systems Ltd., Oxford, UK) motion capture system is a commonly used motion capture system that can export data files in C3D format. During the experiment, this system was equipped with six cameras to capture and store the kinematic data of the reflective markers at a frequency of 100 Hz. In the experiment of capturing the rotation a knee joint, four subjects (two males and two females with an average age of 23.25 years and an average height of 1.70 m) were selected who could freely flex and extend their knee joint in a sitting position; their information is shown in Table 1. Five markers were set on each subject, with four markers fixed on the greater trochanter, lateral condyle, upper tibia, and lateral ankle of the subjects and the fifth marker placed in the area where the seat was in contact with the ground. At the beginning of the experiment, the subjects were in a sitting position, the thighs were parallel to the ground, and the knee joint began to flex and extend, completing the process of lifting and retracting the knee joint from the foot touching the ground to the lower leg parallel to the ground, then repeating the best process multiple times. The Human Ethics Review Committee of Bethune First Hospital of Jilin University (No.2023-312) approved this experimental protocol. Written and oral explanations of the test procedures were provided, and the written consent of the subjects was obtained before the test. The knee joint extension experiment of Subject 1 is shown in Figure 2.

## 4. Experimental Results

**Figure 3:**
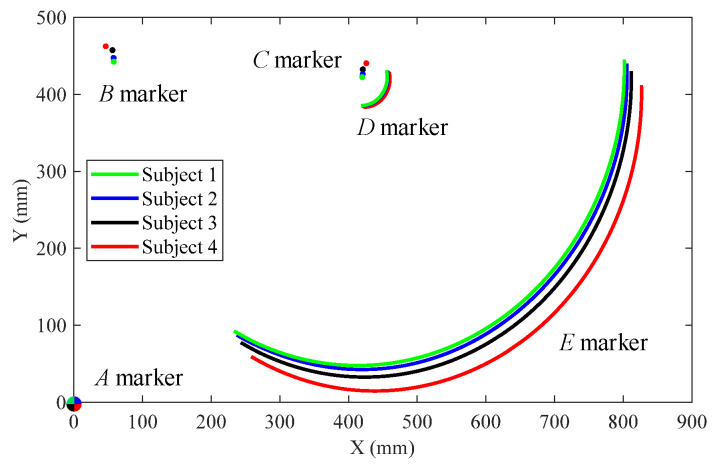
Trajectories of the markers on the four subjects.

For this paper, we only recorded the motion trajectory of the marker point on the sagittal plane of the human body. The global coordinate system *S*_0_(*O*_0_−*x*_0_*y*_0_) was fixed on marker *A* on the ground and the local coordinate system *S*_1_(*O*_1_−*x*_1_*y*_1_) was fixed on marker *C*, as shown in Figure 2. The absolute trajectories of markers *B*, *C*, *D*, and *E* with respect to the ground are shown in Figure 3.

Based on the motion trajectories of each marker in Figure 3, each parameter in the RPR biomimetic knee joint model was calculated as follows:

(2) $$\left\{\begin{array}{c} \alpha_{i}=\left\{\begin{array}{c} -\arctan\frac{Y_{Di}-Y_{Ci}}{X_{Di}-X_{Ci}}+\arctan\frac{Y_{Ci}-Y_{Bi}}{X_{Ci}-X_{Bi}}\;X_{Ci}<X_{Di}\\ -\arctan\frac{Y_{Di}-Y_{Ci}}{X_{Di}-X_{Ci}}+\arctan\frac{Y_{Ci}-Y_{Bi}}{X_{Ci}-X_{Bi}}+\pi \;X_{Ci}\geq X_{Di} \end{array}\right.\\ \gamma_{i}=\left\{\begin{array}{c} -\arctan\frac{Y_{Ei}-Y_{Di}}{X_{Ei}-X_{Di}}+\arctan\frac{Y_{Ci}-Y_{Bi}}{X_{Ci}-X_{Bi}}\;X_{Di}<X_{Ei}\\ -\arctan\frac{Y_{Ei}-Y_{Di}}{X_{Ei}-X_{Di}}+\arctan\frac{Y_{Ci}-Y_{Bi}}{X_{Ci}-X_{Bi}}+\pi \;X_{Di}\geq X_{Ei} \end{array}\right.\\ \beta_{i}=\gamma_{i}-\alpha_{i}\\ L_{i}=\sqrt{{\left(X_{Di}-X_{Ci}\right)}^{2}+{\left(Y_{Di}-Y_{Ci}\right)}^{2}} \end{array}\right.,$$

where *X_Bi_*, *Y_Bi_*, *X_Ci_*, *Y_Ci_*, *X_Di_*, *Y_Di_*, *X_Ei_*, and *Y_Ei_* (*i* = 1, 2, 3, 4) are the horizontal and vertical coordinate values of markers *B*, *C*, *D*, and *E* of the *i*-th experimental subject in the local coordinate system *S*_1_, while *α_i_*, *β_i_*, *L_i_* (*i* = 1, 2, 3, 4) are the characteristic parameters of each motion pair in the RPR model corresponding to the *i*-th experimental subject and *γ_i_* (*i* = 1, 2, 3, 4) are the knee joint angles corresponding to the *i*-th experimental subject.

**Figure 4:**
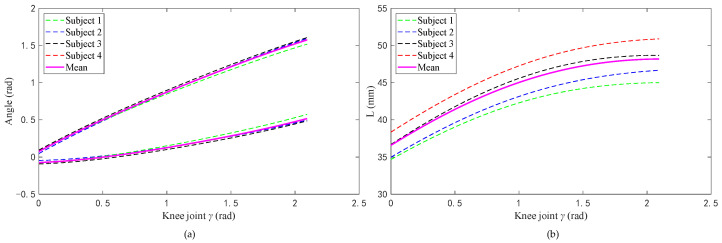
Relationship between kinematic pairs in the RPR model and the knee joint angle value: (**a**) relationship between the angles of two rotating pairs and the knee joint angle and (**b**) relationship between the value of the moving pair and the knee joint angle value.

Figure 4a shows the relationship between the angle (*α_i_*, *β_i_*) of the two rotating pairs in the RPR model and the corresponding angle of the knee joint during knee joint extension. Figure 4b shows the relationship between the length of the moving pair and the corresponding knee joint angle during knee joint extension.

## 5. Characteristic Structural Design of the Knee Orthosis

**Figure 5:**
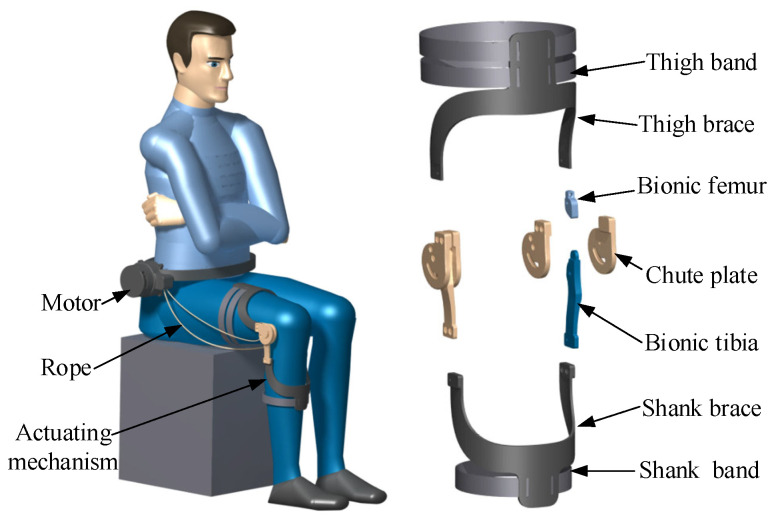
Human–machine interaction diagram and the parts of the developed adaptive knee joint orthosis.

In this section part, an adaptive knee joint orthosis for biomimetic motion assistance containing dual cams is designed based on the RPR biomimetic knee joint model. Here, biomimetic motion assistance means that the motion mode of the orthosis is the same as that of the knee joint. The pitch curve shapes of the two cams are designed based on the screw theory. The orthosis consists of a driving motor, transmission rope, and actuating mechanism. The actuating mechanism includes a thigh band, thigh brace, biomimetic femur, chute plate, biomimetic tibia, shank brace, and shank band, as shown in Figure 5.

The thigh brace and thigh band as well as the shank brace and shank band are components used for binding and fixing the orthosis to the human body. Every two chute plates are wrapped to fix the biomimetic femur and biomimetic tibia; the lower end of biomimetic femur and the upper end of biomimetic tibia are in specific shapes and mesh with each other to achieve rolling, forming a biomimetic knee joint rotation actuating mechanism. As a transmission component, the rope is initially fixed on the driving motor to receive power, which is then transmitted to the actuating mechanism through the involved path, driving the knee joint to undergo biomimetic rotation.

In order to further simplify the shape of each component and achieve biomimetic rotation of the knee joint, the lower end of the biomimetic femur and the upper end of the biomimetic tibia are engaged in a nonlinear curve to achieve variable center rotation. In the design process, it is assumed that two cams were fixed at the lower end of the biomimetic femur and the upper end of the biomimetic tibia and were meshed together to achieve rolling. The trajectory of the rotation center is determined by the pitch curve shape of the cams. This section presents the design of the pitch curve shape of the cam based on the screw theory to ensure the biomimetic rotation of the knee joint mechanism [22].

**Figure 6:**
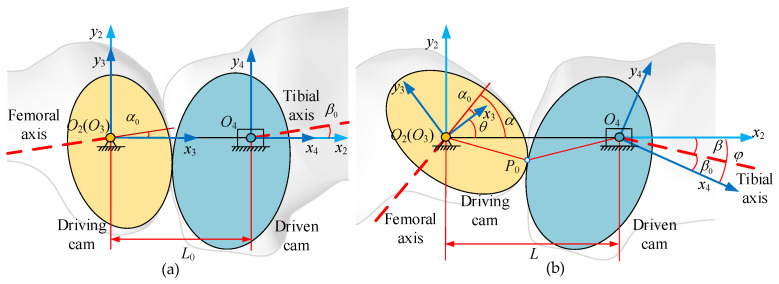
Pitch curve of the meshing cams, showing the schematic diagram of the meshing relationship between the two cams: (**a**) the position of the two cams when the knee joint is at 0 degrees and (**b**) the position of the two cams when the knee joint is at any angle.

The cam at the lower end of biomimetic femur is defined as the driving cam, while the cam at the upper end of biomimetic tibia is defined as the driven cam. Figure 6 shows the coordinate systems and rotation angles of the two cams. Note that *z*-axes of all coordinate frames are omitted, as they are along the same direction and normal to the plane of motion.

The rotation angles of the driving cam and the driven cam can be calculated as follows:

(3) $$\left\{\begin{array}{c} \theta =\alpha -\alpha_{0}\\ \varphi =\beta -\beta_{0} \end{array}\right.,$$

where *α*_0_ is the angle between the center line of the two cams and the femoral axis when the knee joint angle is 0. Then, as the knee joint angle changes, the center line between the two cams and the femoral axis also changes, defined as *α*. *θ* is the difference between *α* and *α*_0_, which is also the rotation angle of the driving cam. Similarly, *β*_0_ and *β* are the angle between the central line of the two cams and the tibia axis when the knee joint angle is 0 or any rotation value, while *φ* is the difference between *β* and *β*_0_, which is also the rotation angle of the driven cam. Finally,

(4) $$r\left(\theta \right)=\frac{\frac{d\varphi }{dt}}{\frac{d\theta }{dt}}=\frac{\frac{d\beta }{dt}}{\frac{d\alpha }{dt}}=\frac{\frac{d\beta }{d\gamma }\cdot \frac{d\gamma }{dt}}{\frac{d\alpha }{d\gamma }\cdot \frac{d\gamma }{dt}}=\frac{\frac{d\beta }{d\gamma }}{\frac{d\alpha }{d\gamma }},$$

where *r*(*θ*)is the function of the transmission ratio about the rotation angle of the driving cam, *t* is the time, and *γ* is the angle of the knee joint.

The relationship of the distance between the centers of the two cams and the rotation angle of the driving cam can be calculated as follows:

(5) $$\left\{\begin{array}{l} L=h(\theta )\\ j\left(\theta \right)=\mathrm{dL}/d\theta \end{array}\right..$$

The coordinate frame *C*_2_ (*O*_2_-*x*_2_*y*_2_*z*_2_) is attached to the base with the *z*_2_-axis along the rotating axis of the driving cam.

The coordinate frame *C*_3_ (*O*_3_-*x*_3_*y*_3_*z*_3_) is connected to the driving cam. The rotating angle of the driving cam *θ* is the angle between the reference frame *C*_3_ and *C*_2_. The coordinate transformation matrix from *C*_2_ to *C*_3_ is as follows:

(6) $$M_{32}\left(\theta \right)=\left[\begin{array}{cccc} \cos\theta & \sin\theta & 0 & 0\\ -\sin\theta & \cos\theta & 0 & 0\\ 0 & 0 & 1 & 0\\ 0 & 0 & 0 & 1 \end{array}\right].$$

The coordinate frame *C*_4_(*O*_4_-*x*_4_*y*_4_*z*_4_) is fixed on the driven gear. Its angle relative to *C*_2_ is the rotating angle of the driven cam *φ*, while its position relative to *C*_2_ is the center distance *L*. The coordinate transformation matrix from *C*_2_ to *C*_4_ is as follows:

(7) $$M_{42}\left(\theta \right)=\left[\begin{array}{cccc} \cos\varphi & -\sin\varphi & 0 & -h\left(\theta \right)\cos\varphi \\ \sin\varphi & \cos\varphi & 0 & -h\left(\theta \right)\sin\varphi \\ 0 & 0 & 1 & 0\\ 0 & 0 & 0 & 1 \end{array}\right].$$

According to the above definition, the screws of the driving cam and driven cam in the coordinate frame *C*_2_ are as follows:

(8) $$\left\{\begin{array}{l} S_{1}=\left[s_{1};\lambda_{1}\right]={\left[0,0,1;0,0,0\right]}^{T}\\ S_{2}=\left[s_{2}\left(\theta \right);\lambda_{2}\left(\theta \right)\right]\\ s_{2}(\theta )={\left[\begin{array}{ccc} 0 & 0 & r\left(\theta \right) \end{array}\right]}^{T}\\ \lambda_{2}\left(\theta \right)={\left[\begin{array}{ccc} j\left(\theta \right) & 0 & 0 \end{array}\right]}^{T}+{\left[\begin{array}{ccc} h\left(\theta \right) & 0 & 0 \end{array}\right]}^{T}\times s_{2}\left(\theta \right)={\left[\begin{array}{ccc} j\left(\theta \right) & -h\left(\theta \right)r\left(\theta \right) & 0 \end{array}\right]}^{T} \end{array}\right.,$$

where S_1_ and S_2_ are the screws of the driving cam and driven cam, respectively, s_1_ and *λ*_1_ are the main vector and dual vector of the screw of the driving cam, respectively, and s_2_(*θ*) and *λ*_2_(*θ*) are the main vector and dual vector of the screw of driven cam, respectively.

According to the theorem of three axes, the instantaneous screw between the driving cam and the driven cam can be calculated as follows:

(9) $$S_{\mathrm{is}}\left(\theta \right)=S_{2}-S_{1}=\left[s_{\mathrm{is}}\left(\theta \right);\lambda_{\mathrm{is}}\left(\theta \right)\right]={\left[\begin{array}{c} \begin{array}{ccc} 0 & 0 & r\left(\theta \right)-1 \end{array}\\ \begin{array}{ccc} j\left(\theta \right) & -r\left(\theta \right)h\left(\theta \right) & 0 \end{array} \end{array}\right]}^{T},$$

where S_is_(*θ*) is the screw between the driving and driven cams, while s_is_(*θ*) and λ_is_ (*θ*) are the main vector and dual vector of the screw between the driving and driven cams.

The position vector of the instantaneous screw axis can be obtained as follows:

(10) $$p_{\mathrm{is}}\left(\theta \right)=\frac{s_{\mathrm{is}}\left(\theta \right)\times \lambda_{\mathrm{is}}\left(\theta \right)}{s_{\mathrm{is}}\left(\theta \right)\cdot s_{\mathrm{is}}\left(\theta \right)}={\left[\begin{array}{ccc} \frac{h\left(\theta \right)r\left(\theta \right)}{r\left(\theta \right)-1} & \frac{j\left(\theta \right)}{r\left(\theta \right)-1} & 0 \end{array}\right]}^{T},$$

where p_is_(*θ*) is the position vector of the instantaneous screw axis.

The pitch curve matrixes of the driving cam and the driven cam are calculated as follows:

(11) $$\left\{\begin{array}{l} T_{1}=M_{32}\left(\theta \right)\cdot \left[\begin{array}{c} p_{\mathrm{is}}\left(\theta \right)\\ 1 \end{array}\right]=\frac{1}{r\left(\theta \right)-1}\left[\begin{array}{c} j\left(\theta \right)\sin\theta +h\left(\theta \right)r\left(\theta \right)\cos\theta \\ j\left(\theta \right)\cos\theta -h\left(\theta \right)r\left(\theta \right)\sin\theta \\ 0\\ 1 \end{array}\right]\\ T_{2}=M_{42}\left(\varphi \right)\cdot \left[\begin{array}{c} p_{\mathrm{is}}\left(\theta \right)\\ 1 \end{array}\right]=\frac{1}{r\left(\theta \right)-1}\left[\begin{array}{c} j\left(\theta \right)\sin\varphi +h\left(\theta \right)\cos\varphi \\ j\left(\theta \right)\cos\varphi -h\left(\theta \right)\sin\varphi \\ 0\\ 1 \end{array}\right] \end{array}\right.,$$

where T_1_ is the pitch curve matrix of the driving cam and T_2_ is the pitch curve matrix of the driven cam.

**Figure 7:**
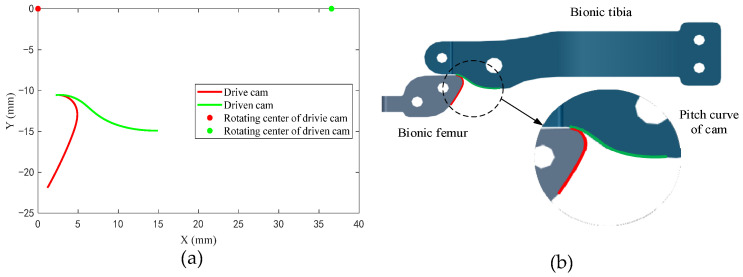
Design result: (**a**) pitch curve of the meshing cams and (**b**) meshing of the biomimetic femur and biomimetic tibia.

The first, second, and third rows of the pitch matrix (T_1_ and T_2_) are the coordinate values of the pitch curve in the reference frame *C*_2_ (*O*_2_−*x*_2_*y*_2_*z*_2_). Figure 7a shows the projection of the pitch curve of the driving cam and driven cam on the *x*_2_*y*_2_ plane of the coordinate system *C*_2_ (*O*_2_−*x*_2_*y*_2_*z*_2_). Figure 7b shows the meshing assembly relationship the biomimetic femur and biomimetic tibia.

## 6. Light Weight of the Orthosis

The adaptive knee joint orthosis is designed for patients who require biomimetic motion assistance. Therefore, while ensuring structural safety, it is necessary to minimize the weight quality of the structure as much as possible. The main load-bearing components of the actuating mechanism are thin-walled components. The mass of the components can be reduced by size optimization [23]. The initial wall thickness of each part is 5 mm, and only the maximum bearing state of the equipment when the knee joint angle is 0 is analyzed. To ensure user comfort during the device assistance process, the maximum displacement of the components is set to be less than 10 mm. The material is steel, with a density of 7850 kg/m^3^, an elastic modulus of 210 GPa, a Poisson’s ratio of 0.3, and a yield limit of 355 MPa. The safety factor is set to 1.2, and the allowable stress and displacement of the equipment are 295.8 MPa and 8.2 mm, respectively.

### 6.1. Loading in the Extreme Condition

**Figure 8:**
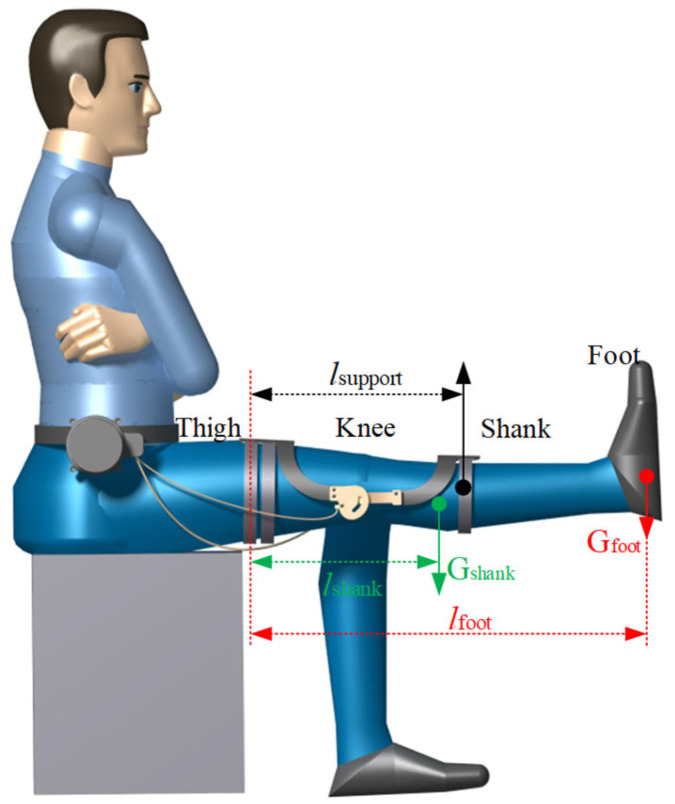
The maximum load-bearing state of orthotics in human–computer interaction.

The load of the actuating mechanism in the orthosis is related to the size of the user’s lower limbs, the quality of the user’s lower limb, and the human–machine states. The operating conditions of the orthosis were set as follows: the user was seated on a chair with both ends of the orthosis fixed to the thighs and shank, while the thighs remained stationary and the rope drove the actuating mechanism to assist the shank to extend. When the actuating mechanism assisted the shank to rise to the horizontal state, the gravity of the shank and the foot was all borne by the orthosis, which is the maximum load-bearing state of the orthosis, as shown in Figure 8. It is necessary to know the size and mass of the thigh, shank, and foot of the user before calculating the supporting force of the actuating mechanism in the maximum bearing state. The user of the equipment is assumed to be an adult male (height 1.70 m, weight 74 kg). According to the human dimensions of Chinese adults and the inertial parameters of a Chinese adult human body [24,25], the size and mass of the user’s thigh, shank and foot can be obtained as shown in Table 2.

As shown in Figure 8, in the maximum load-bearing condition the thigh brace of the actuating mechanism is fixed on the thigh, then the shank brace of the actuating mechanism is driven to provide lifting force for the shank which is equal to the reaction force from the shank to the shank brace, calculated as 38.3 N by Equation (12):

(12) $$F=\frac{G_{shank}\cdot l_{shank}+G_{foot}\cdot l_{foot}}{l},$$

where *F* is the lifting force, *G_shank_* is the gravity of the shank, *G_foot_* is the gravity of the foot, *l* is the horizontal distance from the center of rotation of the lifting force, *l_shank_* is the horizontal distance from the center of gravity of the shank to the center of rotation, and *l_foot_* is the horizontal distance from the center of gravity of the shank to the center of rotation.

### 6.2. Finite Element Analysis of the Mechanical Structure

**Figure 9:**
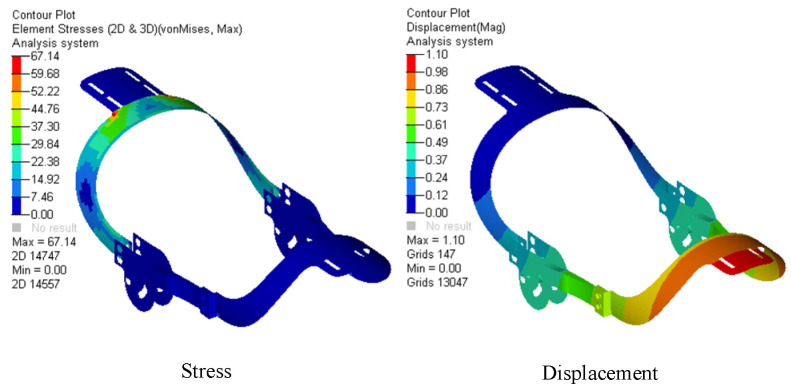
Finite element analysis results of equipment with initial wall thickness.

Based on the working conditions of the model in Figure 8, we established the finite element model of the actuating mechanism and conducted finite element analysis. The finite element model contained 8508 elements and 8653 nodes. The results are shown in Figure 9. It can be seen the maximum stress and maximum displacement of the model are less than the allowable stress and allowable displacement, meaning that the weight of the mechanism can be lightened further.

The optimization results are shown in Table 3. Size optimization was performed on the model using Equation (13), where the wall thickness of the parts is the independent variable, the allowable stress of 295.8 MPa and allowable displacement of 8.3 mm are the constraint conditions, and the minimum mass is the objective function:

(13) $$\left\{\begin{array}{l} \min M=\sum_{i=1}^{n}m_{j},\;j=1,2,3\\ m_{j}=f\left(x_{k}\right),\;k=1,2,3\;\\ \sigma_{\max}\leq 295.8\;\mathrm{MPa}\;\\ d_{\max}\leq 8.3\;\mathrm{mm}\; \end{array}\right.$$

where *M* is the total mass of the mechanical structure, *m_j_* (*j* = 1, 2, 3) are the masses of thigh brace, shank brace, and chute plate, *f* is the mass function, *x_k_* (*k* = 1, 2, 3) are the wall thicknesses of the thigh brace, shank brace, and chute plate, which are design variables, *δ*_max_ is the maximum stress, and *d*_max_ is the maximum displacement.

**Figure 10:**
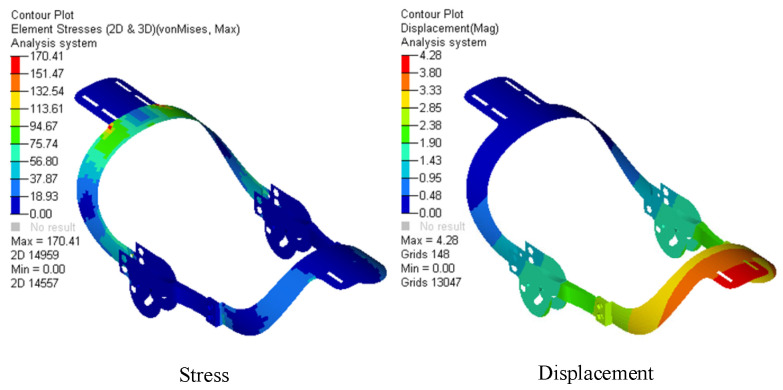
Finite element analysis results of the mechanism after size optimization.

The wall thicknesses resulting from size optimization were reassigned to the mechanical structure, then the stress and displacement were obtained, as shown in Figure 10. The results show that the stress is less than 295.8 MPa and the displacement is less than 8.2 mm, which meets the design requirements. The total weight of the mechanism was reduced from 1.96 kg to 1.16 kg, a decrease of 40.8%, meeting the goal of light weight.

## 7. Verification of the Prototype

**Figure 11:**
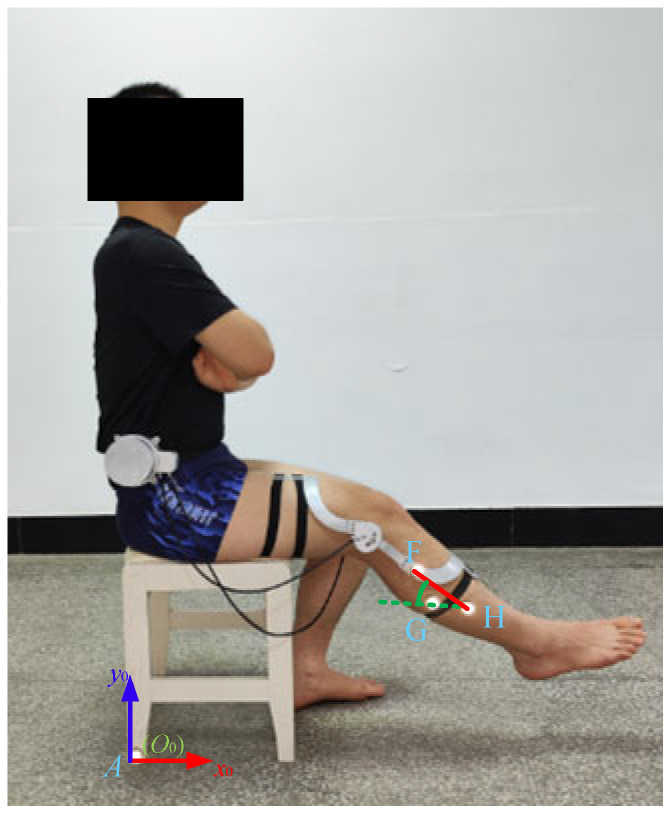
Verification experiment of the prototype of the knee joint orthosis for biomimetic motion assistance (with reflective markers on points A, F, H, and G).

**Figure 12:**
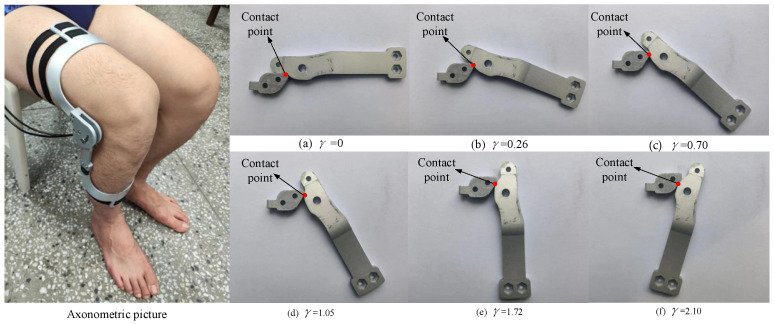
The axonometric diagram of the prototype and movement of the biomimetic tibia and biomimetic femur during the experiment; *γ* represents the knee joint angles.

**Figure 13:**
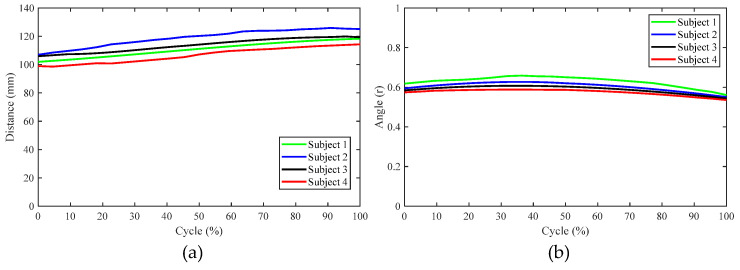
The results of the verification experiment: (**a**) the distance between markers *F* and *H* during knee extension and (**b**) the value of *∠FGH* during knee extension.

In order to verify the usability of the aforementioned design scheme, a prototype orthosis was made to assist the knee joint in biomimetic extension. To verify the biomimetic performance of orthosis, four subjects were recruited to participate in the validation experiment, as shown in Figure 11. After the subject wore the orthosis and sat in the chair, the motor (DC servo motor, Rated voltage is 24 V) was activated to drive knee joint extension and the trajectories of the reflective markers on the thigh and shank during the knee joint extension process were recorded using the Vicon motion capture system. This experimental protocol was approved by the Human Ethics Review Committee of Bethune First Hospital of Jilin University (No. 2023-312). Written and oral explanations of the test procedures were provided and the written consent of the subjects was obtained before the test. The axonometric diagram of the prototype and the movement of the biomimetic tibia and biomimetic femur during the experiment are shown in Figure 12. During the experiment, the motion trajectories of the *F*, *G*, and *H* positions during the knee joint movement of the four subjects were obtained. Further calculation revealed that the distance between points *F* and *H* and the angle ∠*FGH* were almost unchanged, as shown in Figure 13, indicating that the relevant components in the orthosis did not deviate from the attached limb segments during knee joint extension and that the orthosis was relatively stationary with respect to the thigh and shank. Thus, the orthosis can perform the expected assistance according to the biomimetic motion requirements of the knee joint.

## 8. Conclusions

An adaptive knee joint orthosis with a variable rotation center was designed. The optimized mechanism realizes biomimetic motion through two engaged cams with specific pitch curve shapes, helping patients to extend their knees. The weight of the equipment was lighted using finite element analysis method to reduce the burden of patients during the process of rehabilitation and reduce the manufacturing cost of the equipment. Finally, the functioning of a prototype developed to assist the knee joint with desired biomimetic motion was verified.

In future work, we intend to increase the number of subjects to make up for there being only four subjects in this paper and to ensure that the orthosis is suitable for a wider range of people. Thus, we will take into account the characteristics of different patients in order to further improve the equipment to meet individual needs. In addition, we will further reduce the mass of the equipment, improve its portability, and reduce manufacturing costs by exploring other materials with better mechanical properties.

## Figures and Tables

**Table 1:** Information about the four subjects.

Subjects	Ages	Sex	Height (m)	Heathy	Range of Motion of Knee Joint (r)
1	23	Male	1.69	Yes	0–2.09
2	25	Male	1.76	Yes	0–2.09
3	21	Female	1.65	Yes	0–2.09
4	24	Female	1.70	Yes	0–2.09

**Table 2:** Mass and size of the user’s lower limb segments.

Body Segments	Weight (kg)	Size (mm)
Thigh	10.5	470
Shank	2.72	373

**Table 3:** Size optimization results of the equipment.

Part	Original Thicknesses (mm)	Range of Thicknesses (mm)	Optimized Thicknesses (mm)	Final integer Thicknesses (mm)
Thigh brace	5	1–10	2.54	3
Shank brace	5	1–10	1.05	2
Chute plate	5	1–10	1	1

## Data Availability

Data are unavailable due to privacy or ethical restrictions.
